# H_2_O_2_ and Ca^2+^ Signaling Crosstalk Counteracts ABA to Induce Seed Germination

**DOI:** 10.3390/antiox11081594

**Published:** 2022-08-17

**Authors:** Mengjie Cheng, Yanliang Guo, Qing Liu, Sanwa Nan, Yuxing Xue, Chunhua Wei, Yong Zhang, Feishi Luan, Xian Zhang, Hao Li

**Affiliations:** 1 College of Horticulture, Northwest A&F University, Yangling 712100, China; 2 College of Horticulture and Landscape Architecture, Northeast Agricultural University, Harbin 150000, China

**Keywords:** hydrogen peroxide, calcium signal, abscisic acid, gibberellic acid, seed germination

## Abstract

Seed germination is a critical stage and the first step in the plant’s life cycle. H_2_O_2_ and Ca^2+^ act as important signal molecules in regulating plant growth and development and in providing defense against numerous stresses; however, their crosstalk in modulating seed germination remains largely unaddressed. In the current study, we report that H_2_O_2_ and Ca^2+^ counteracted abscisic acid (ABA) to induce seed germination in melon and *Arabidopsis* by modulating ABA and gibberellic acid (GA_3_) balance. H_2_O_2_ treatment induced a Ca^2+^ influx in melon seeds accompanied by the upregulation of *cyclic nucleotide-gated ion channel* *(CNGC) 20*, which encodes a plasma membrane Ca^2+^-permeable channel. However, the inhibition of cytoplasmic free Ca^2+^ elevation in the melon seeds and *Arabidopsis* mutant *atcngc20* compromised H_2_O_2_-induced germination under ABA stress. CaCl_2_ induced H_2_O_2_ accumulation accompanied by the upregulation of *respiratory burst oxidase homologue* *(RBOH) D* and *RBOHF* in melon seeds with ABA pretreatment. However, inhibition of H_2_O_2_ accumulation in the melon seeds and *Arabidopsis* mutant *atrbohd* and *atrbohf* abolished CaCl_2_-induced germination under ABA stress. The current study reveals a novel mechanism in which H_2_O_2_ and Ca^2+^ signaling crosstalk offsets ABA to induce seed germination. H_2_O_2_ induces Ca^2+^ influx, which in turn increases H_2_O_2_ accumulation, thus forming a reciprocal positive-regulatory loop to maintain a balance between ABA and GA_3_ and promote seed germination under ABA stress.

## 1. Introduction

Seed germination is the first and vital step in a plant’s life cycle [1]. This physiological process starts with water absorption by dry seeds and ends with radicle emergence, during which the imbibed seeds shift from a quiescent state to active metabolism [2]. The transition from seed dormancy to germination involves simultaneous seed reserve mobilization and seed coat rupture, which cumulate in radicle emergence and subsequent seedling establishment [3,4]. Seed germination is a complex process that depends on multiple environmental factors, such as temperature, water availability, light, and oxygen, as well as certain intrinsic factors such as phytohormones [5]. Both poor seed quality and sowing conditions adversely affect seed germination and subsequent crop establishment, health, and yield [6].

Abscisic acid (ABA) and gibberellic acid (GA) are two central hormones that transduce environmental information and play antagonistic roles in regulating seed germination [7]. In fact, seed dormancy or germination largely depends on the dynamic balance of ABA and GA [8]. ABA promotes seed dormancy but inhibits seed germination. Knockout of ABA biosynthesis- or signaling-related genes promoted seed germination and reduced seed dormancy [9]. However, the mutation of ABA catabolic enzymes or overexpression of ABA biosynthetic enzymes delays seed germination but prolongs seed dormancy [2,10]. Conversely, GA breaks seed dormancy and promotes seed germination by activating the growth potential of embryos and weakening the embryo surrounding tissues [11].

In plants, reactive oxygen species (ROS), such as hydroxyl radicals (OH^.^), superoxide (O_2_^−^), and hydrogen peroxide (H_2_O_2_), play important roles in regulating seed dormancy or germination [12,13]. The controlled germination of ROS during seed imbibition can oxidize a subset of biomolecules such as mRNAs, nucleic acids, amino acids, and proteins, resulting in adequate cell functions that trigger seed germination [14]. In particular, H_2_O_2_ generated by *respiratory burst oxidase homologues* (*RBOHs*)-encoded NADPH oxidase is a critical secondary signaling molecule that regulates ABA and GA balance and affects their signaling pathways in regulating seed germination [15,16].

In addition to ROS, calcium (Ca^2+^) functions as a vital second messenger and plays an important role in regulating various physiological processes, including seed germination [17,18]. The Ca^2+^ signal functions by eliciting characteristic transient fluctuations of cytoplasmic free Ca^2+^ ([Ca^2+^]_cyt_) concentration through membrane transport proteins-mediated activities of Ca^2+^ influx and efflux [19]. Activated Ca^2+^ influx channels, such as two-pore channels (TPCs), glutamate receptor homolog (GLR) 3.5, and cyclic nucleotide-gated ion channels (CNGCs), induce a transient [Ca^2+^]_cyt_ elevation and then trigger Ca^2+^ signal, which is decoded and relayed to downstream regulators of gene expression by a set of Ca^2+^ sensor proteins [19]. Abundant evidence suggests that the Ca^2+^ signal plays an important role in regulating seed germination by altering ABA and GA signaling [20,21].

Despite abundant studies on the individual role of H_2_O_2_ and Ca^2+^ in regulating seed germination, their potential interaction in modulating ABA/GA balance and subsequent seed germination remains largely unknown. The crosstalk of H_2_O_2_ (a major ROS) and Ca^2+^ regulating plant growth and development and its role as a defense against numerous stresses have been well documented [22]. For example, in plant response to cold exposure, Ca^2+^ signal is rapidly induced and then activates RBOH activity to trigger H_2_O_2_ production, which in turn induces Ca^2+^ transient influx in plant cells, forming a reciprocal positive-regulatory loop [23,24,25]. This raises the possibility that H_2_O_2_ and Ca^2+^ may also interact with each other in modulating seed germination. We demonstrate that the positive crosstalk of H_2_O_2_ and Ca^2+^ promotes seed germination under ABA stress. H_2_O_2_ promotes Ca^2+^ influx, which in turn increases H_2_O_2_ accumulation to regulate ABA and GA balance.

## 2. Materials and Methods

### 2.1. Plant Materials

In this study, melon (*Cucumis melo* L. cv. SSMA) seeds were provided by the Watermelon and Melon Research Group of Northwest A&F University, Yangling, China. Seeds of *Arabidopsis* mutant *atrbohd* (SALK_120299), *atrbohf* (SALK_034674), and *atcngc20* (SALK_074919C) with a Columbia genetic background were obtained from the Arabidopsis Biological Resource Center (https://www.arabidopsis.org/, accessed on 1 June 2018).

### 2.2. Experimental Design

To investigate the role and mechanisms of H_2_O_2_ and Ca^2+^ in counteracting ABA to promote seed germination, the melon or *Arabidopsis* seeds were soaked in double distilled water (as control) or different test solutions for 7 h and then were rinsed three times with double-distilled water. The rinsed melon seeds were incubated at 30 °C in the dark for 7 days in 9 cm diameter Petri dishes containing double-layered rolled filter paper moistened with distilled water. The rinsed *Arabidopsis* seeds were incubated at 21 °C under continuous light at 100 μM m^−2^ s^−1^ for 7 days in 3 cm Petri dishes containing moistened filter papers. Each treatment comprised three replicates. Each replicate consisted of 30 seeds. Seeds were considered germinated when the radicle emerged (1–2 mm). Germination was scored daily as radicle emergence.

Test solutions used to treat melon seeds included ABA (1 mM), ABA (1 mM) + H_2_O_2_ (10 mM), ABA (1 mM) + CaCl_2_ (0.2, 0.5, 1, or 3 mM), ABA (1 mM) + diphenyleneiodonium (DPI, an inhibitor of NADPH oxidases, which produces ROS, 10 μM) [26,27,28], ABH (1 mM ABA + 10 mM H_2_O_2_) + ethylene glycol-bis (2-aminoethylether)-*N,N,N′,N′*-tetraacetic acid (EGTA, a Ca^2+^ chelator, 5 mM) [18,29], ABH + LaCl_3_ (a plasma membrane-located Ca^2+^ channel blocker, 5 mM) [18,29], ACa (1 mM ABA + 1 mM CaCl_2_) + DPI (10 μM). Test solutions used to treat *Arabidopsis* seeds included ABA (0.2 mM), ABA (0.2 mM) + H_2_O_2_ (5 mM), ABA (0.2 mM) + CaCl_2_ (0.2, 0.5, or 1 mM). The concentrations of the different chemicals are referred to Li et al. [30].

**Figure 1:**
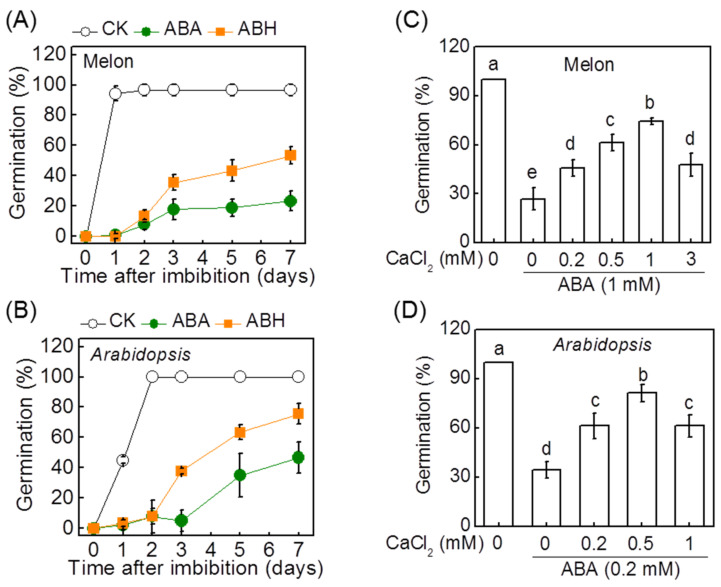
H_2_O_2_ and Ca^2+^ counteract abscisic acid (ABA) to promote seed germination in melon and *Arabidopsis*. (**A**,**B**) The effects of ABA and the combination of ABA and H_2_O_2_ (ABH) on the germination of melon and *Arabidopsis* seeds. (**C**,**D**) The effects of ABA and the combination of ABA and CaCl_2_ (ACa) on the germination of melon and *Arabidopsis* seeds. The melon seeds were presoaked in double-distilled water or test solutions for 7 h and then were incubated at 30 °C for 7 days. In (**C**,**D**), the germination rates were recorded on the seventh day after seed incubation. Data are means ± SD (3 replicates × 30 seeds). The different letters denote a significant difference (*p* < 0.05) according to Tukey’s test.

Notably, the chemical concentrations were higher in this study than those in previous studies. These high concentrations of chemicals were also used to assess their functions in various plant species [27,30]. The chemical concentrations were justified previously by using a range of concentrations, and the optimum concentration was used [30]. Generally, in previous studies, the chemicals such as ABA were added to the incubation medium during seed germination for several days. In the current study, the seeds were soaked with test solutions for 7 h and then rinsed three times with distilled water. Such treatments can increase endogenous chemical content and avoid exogenous chemicals’ constant and complex influence during seed incubation. After ABA (1 mM) pretreatment for 7 h, the endogenous ABA in melon seeds increased from 16.8 to 133.6 ng g^−1^ [30]. Therefore, a high concentration of ABA did not completely inhibit seed germination owing to the short treatment time (see Figure 1).

### 2.3. Net Ca^2+^ Flux Assay

The net Ca^2+^ flux of the seed embryo cells was measured with a Noninvasive Micro-test Technology (NMT) Physiolyzer (Xuyue Science and Technology Company Limited, Beijing, China) following the method of Li et al. [30]. The flux unit is pmol cm^−2^ s^−1^, and the negative and positive values represent Ca^2+^ influx and efflux, respectively.

### 2.4. Analysis of H_2_O_2_

The H_2_O_2_ content in seeds was measured according to the method described by Willekens et al. [31]. In brief, 0.2 g samples were ground in 3 mL of ice-cold HClO_4_ (1 M). After centrifugation at 6000× *g* for 5 min at 4 °C, the supernatant pH was adjusted to 6.0–7.0 with KOH (4 M). The supernatants were further centrifuged at 12,000× *g* for 5 min at 4 °C, passed through an AG1-X8 prepacked column (Bio-Rad, Hercules, CA, USA), and then were eluted with 4 mL ddH_2_O. The sample extract (800 μL) was combined with 400 μL of 100 mM potassium acetate (pH 4.4) containing horseradish peroxidase (0.25 U), 2,2′-azino-di (3-ethylbenzthiazoline-6-sulfonic acid) (4 mM), and 400 μL deionized water. The H_2_O_2_ content was measured at OD_412_.

### 2.5. Analysis of ABA and GA_3_

The plant hormones were extracted as described by Yang et al. [32] with minor modifications. In brief, 0.3 g of frozen seed samples was homogenized in 4 mL of methanol (80%, *v*/*v*) containing 1 mM 2,6-di-*t*-butyl-*p*-cresol as an antioxidant. The homogenates were incubated at 4 °C for 4 h and then were centrifuged at 1000× *g* for 20 min at 4 °C. The obtained supernatants (crude extracts) were filtered through C18 Sep-Park Cartridge (Millipore, Millford, MA, USA), dried under N_2_ (gas) flow, and then were dissolved in 5 mL of 50 mM Tris (pH 8.1) containing 10% (*v*/*v*) methanol, 1 mM MgCl_2_, and 150 mM NaCl. ABA and GA_3_ contents were analyzed with an immunoassay kit (China Agricultural University, Beijing, China) following the manufacturer’s instructions. Colorimetric readings were conducted using a Multimode Plate Reader M200 pro (Tecan, Männedorf, Switzerland).

### 2.6. qRT-PCR Analysis

The total RNA were isolated from the seeds using an RNA extraction kit (Axgen, Union City, CA, USA). After extraction, a DNase Mini Kit (Qiagen, Hilden, Germany) was used to remove residual DNA. Then, total RNA (1 µg per sample) was reverse-transcribed to cDNA using a FastKing RT kit (TIANGEN, Beijing, China). qRT-PCR was conducted on an iCycler Iq TM Multicolor PCR Detection System (Bio-Rad, Hercules, CA, USA) using SYBR^®^ Premix ExTaq^TM^ II (2×) kit (Takara, Tokyo, Japan). The gene-specific primers were designed according to the EST sequences (http://cucurbitgenomics.org/, accessed on 1 March 2018): 5′-TTGGTGCTGGCGAATTGGTTGA-3′ and 5′-ATGATCTGAGGCAGCGGCAAA-3′ for *CmCNGC20* (MELO3C001941); 5′-AGTGAGTGACAGCCGAGTTCTAAGT-3′ and 5′-CTGCTCTGTGACGGTATTGGATGAA-3′ for *CmRBOHD* (MELO3C026754); 5′-GCACGAGTTGAAGGCTGAGTTGA-3′ and 5′-GGAATCCATCCTTGGCGAGCTTATC’ for *CmRBOHF* (MELO3C005718); and 5′-ATTCTTGCATCTCTAAGTACCTTCC-3′ and 5′-CCAACTAAAGGGAAATAACTCACC-3′ for *CmActin* (MELO3C008032). *CmActin* was used as the internal control genes [30]. The relative expression of mRNA was calculated as described previously [33].

### 2.7. Statistical Analysis

The experiment was a completely randomized design with three independent replications. Each replicate included 30 seeds. Analysis of variance (ANOVA) was used to test for significance, and significant differences among treatments were determined using Tukey’s test at the *p* < 0.05 level.

## 3. Results

### 3.1. H_2_O_2_ and Ca^2+^ Counteract ABA to Induce Seed Germination

Exogenous ABA pretreatment remarkably delayed germinating time and severely reduced germination rates of both melon and *Arabidopsis* seeds (Figure 1). However, exogenous H_2_O_2_ or CaCl_2_ pretreatment attenuated the ABA-induced inhibition of seed germination in melon and *Arabidopsis* seeds. The most effective concentration of CaCl_2_ was 1 mM and 0.5 mM for melon and *Arabidopsis* seeds, respectively. The promoting effect of CaCl_2_ on seed germination was attenuated with CaCl_2_ concentrations both lower and higher than the optimum concentration. For example, the germination rates of melon seeds pretreated with ABA (1 mM) + H_2_O_2_ (10 mM) and ABA (1 mM) + CaCl_2_ (1 mM) were 53.3% and 74.4%, respectively, far higher than that of seeds with ABA pretreatment.

### 3.2. Ca^2+^ Signal Is Involved in H_2_O_2_-Induced Seed Germination

**Figure 2:**
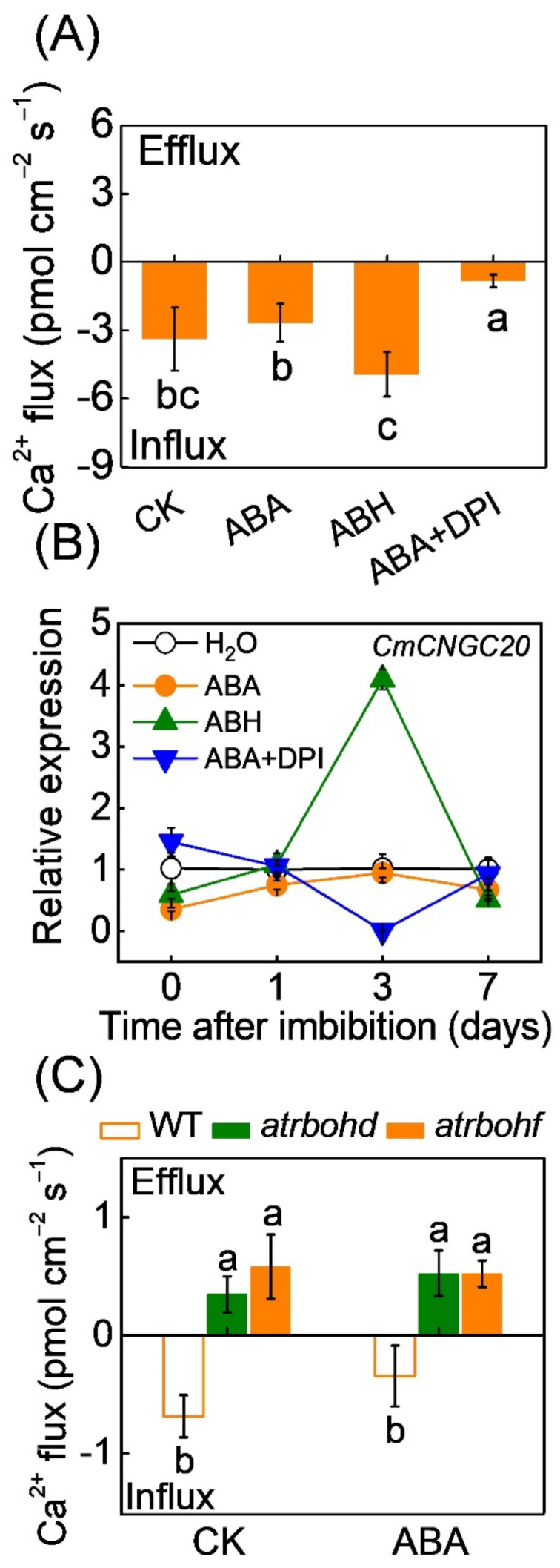
The effects of H_2_O_2_ on Ca^2+^ flux in seeds under ABA stress. (**A**,**B**) Ca^2+^ flux and *CmCNGC20* expression in melon seeds pre-treated with ABA, a combination of ABA and H_2_O_2_ (ABH), and a combination of ABA and diphenyleneiodonium (DPI, an inhibitor of NADPH oxidases, which produces H_2_O_2_). (**C**) Ca^2+^ flux in seeds of wild-type (WT) *Arabidopsis* and *atrbohd* and *atrbohf* mutants under normal conditions and ABA stress. In (**A**,**C**), dry melon seeds or *Arabidopsis* were soaked in double distilled water or test solutions for 7 h and then were used to analyze the changes in Ca^2+^ flux. In (**B**), melon seeds were treated as that in Figure 1. Data are means ± SD (3 replicates × 30 seeds). The different letters denote a significant difference (*p* < 0.05) according to Tukey’s test.

To investigate whether the Ca^2+^ signal participates in H_2_O_2_-induced seed germination under ABA stress, we firstly analyzed the effects of H_2_O_2_ and its deficiency on Ca^2+^ flux. As shown in Figure 2, the ABA pretreatment had very little effect on the net flux of Ca^2+^ in both melon and *Arabidopsis* seeds. Compared to ABA, ABH (ABA+H_2_O_2_) pretreatment induced Ca^2+^ influx, accompanied by an increase in the transcript level of *CmCNGC20*, a gene encoding a plasma membrane Ca^2+^-permeable channel (Figure 2A,B). However, ABA+DPI (an inhibitor of NADPH oxidase-mediated ROS generation, 10 μM) decreased Ca^2+^ influx and downregulated the expression of *CmCNGC20*. Consistently, *atrbohd* and *atrbohf* mutation in *Arabidopsis* seeds inhibited Ca^2+^ influx under both control conditions and ABA stress (Figure 2C). These results suggest that RBOH-dependent ROS mediates Ca^2+^ influx during seed germination under ABA stress.

**Figure 3:**
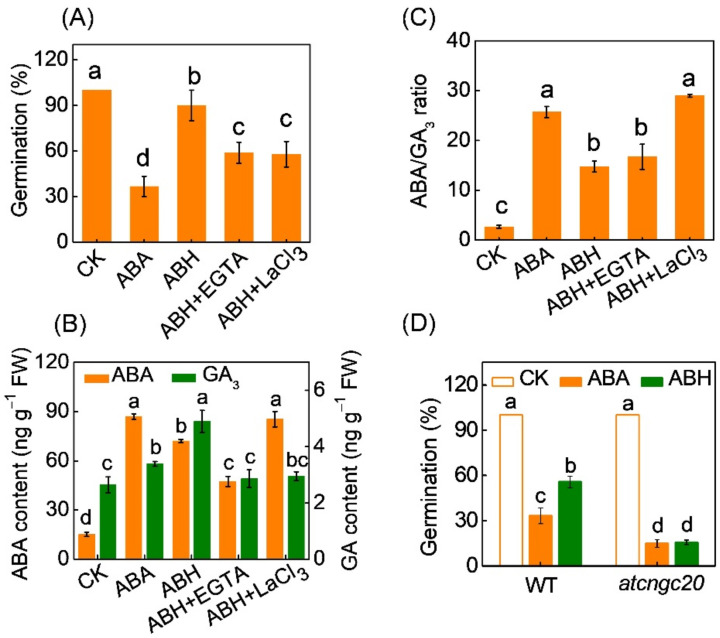
The role of Ca^2+^ in H_2_O_2_ counteracting ABA to promote seed germination. (**A**) The germination rates of melon seeds treated with ABA, a combination of ABA and H_2_O_2_ (ABH), a combination of ABH and EGTA (a Ca^2+^ chelator), and a combination of ABH and LaCl_3_ (a plasma membrane-located Ca^2+^ channel blocker). (**B**,**C**) ABA and GA_3_ content and ABA/GA_3_ ratio in melon seeds treated with ABA, ABH, ABH+EGTA, and ABH+LaCl_3_. ABA and GA_3_ contents were measured on the third day after seed incubation. (**D**) The effects of ABA and ABH on the germination rates of seeds in wild-type (WT) *Arabidopsis* and *atcngc20* mutant. In both (**A**,**D**), germination rates were recorded on the seventh day after seed incubation. Data are means ± SD (3 replicates × 30 seeds). The different letters denote significant difference (*p* < 0.05) according to Tukey’s test.

To further investigate the role of Ca^2+^ signals in H_2_O_2_-induced seed germination and ABA/GA_3_ balance under ABA stress, EGTA (a Ca^2+^ chelator) and LaCl_3_ (a plasma membrane-located Ca^2+^ channel blocker) were used to chelate Ca^2+^ and block Ca^2+^ influx, respectively. As shown in Figure 3A, both EGTA and LaCl_3_ inhibited H_2_O_2_-induced seed germination under ABA stress. The germination rates of seeds pretreated with ABA, ABA+H_2_O_2_ (ABH), ABH+EGTA, and ABH+LaCl_3_ were 36.7%, 90.0%, 58.9%, and 57.8%, respectively. EGTA but not LaCl_3_ promoted ABH-induced decrease in ABA level (Figure 3B). However, both EGTA and LaCl_3_ completely compromised the ABH-induced increase of GA level. As a result, LaCl_3_ but not EGTA abolished ABH-decreased ABA and GA_3_ ratio. The ABA/GA_3_ ratio in seeds with ABH+LaCl_3_ pretreatment was 29.0, significantly higher than that (14.7) in seeds with ABH pretreatment. When compared to wild-type *Arabidopsis* seeds, seeds of *atcngc20* mutant showed lower germination rates under ABA stress (Figure 3D). However, *AtCNGC20* deletion attenuated H_2_O_2_-induced seed germination under ABA stress.

### 3.3. H_2_O_2_ Mediates Ca^2+^-Induced Seed Germination

**Figure 4:**
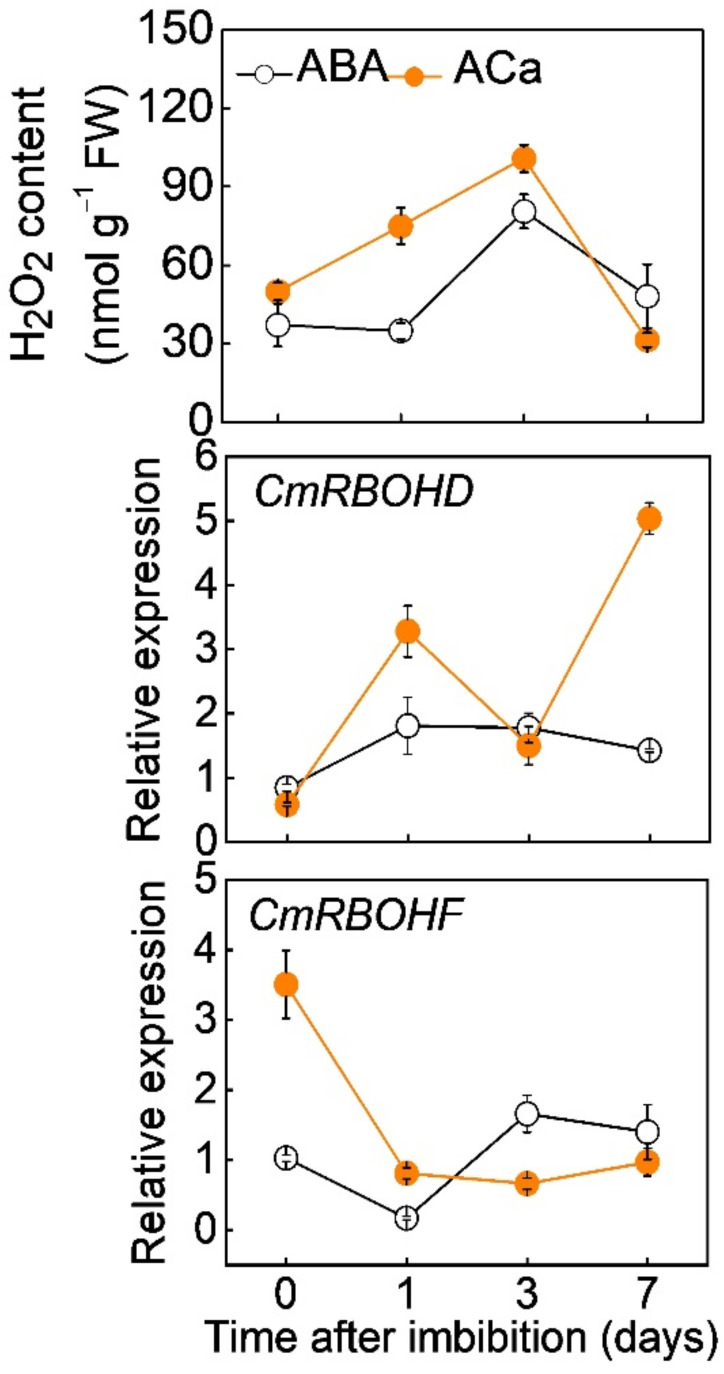
The effects of ABA and combination of ABA and CaCl_2_ (ACa) on the H_2_O_2_ accumulation and the relative expression of *CmRBOHD* and *CmRBOHF* during melon seed incubation. Data are means ± SD (3 replicates × 30 seeds).

To investigate whether H_2_O_2_ was involved in Ca^2+^-induced seed germination under ABA stress, we analyzed the effects of CaCl_2_ on H_2_O_2_ accumulation under ABA stress. During incubation at 30 °C, the H_2_O_2_ level in melon seeds increased by ABA pretreatment from the first day, reached their peaks on the third day, and then declined gradually (Figure 4). CaCl_2_ promoted ABA-induced H_2_O_2_ accumulation. On the 1st and third day of incubation at 30 °C, H_2_O_2_ content in melon seeds pretreated with ABA+CaCl_2_ (ACa) were 115.2% and 25.0% higher, respectively, than that in the seeds pretreated with ABA alone. The expression of *CmRBOHD* was increased by ABA pretreatment, and this increase was promoted by CaCl_2_ on the first and seventh days during seed incubation. The expression of *CmRBOHF* was increased by ABA pretreatment from the third day of incubation. CaCl_2_ upregulated *CmRBOHF* expression in early response to ABA (within one day after seed incubation).

**Figure 5:**
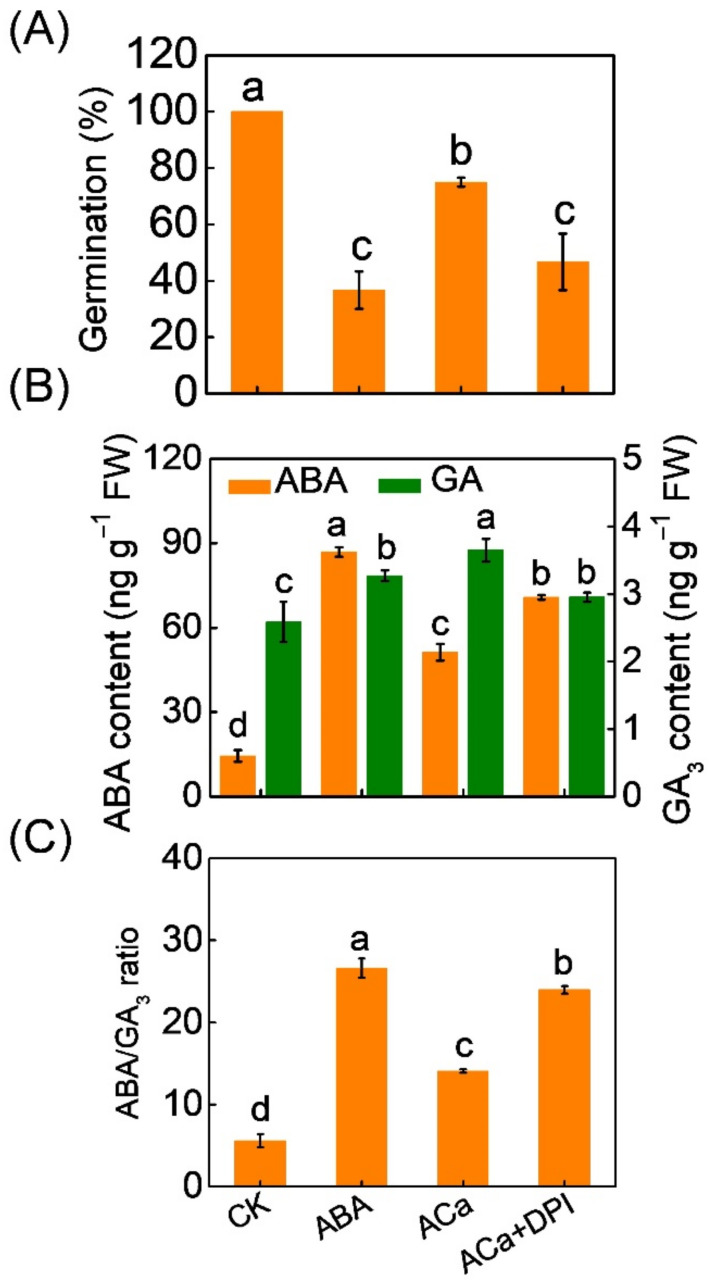
Involvement of H_2_O_2_ in Ca^2+^ counteracting ABA to promote seed germination. (**A**) The germination rate of melon seeds treated with ABA, a combination of ABA and CaCl_2_ (ACa), and a combination of ACa and diphenyleneiodonium (DPI, an inhibitor of NADPH oxidases, which produces ROS). Germination rates were recorded on the seventh day after seed incubation. (**B**,**C**) ABA and GA_3_ content and ABA/GA_3_ ratio in melon seeds treated with ABA, a combination of ABA and CaCl_2_ (ACa), and a combination of ACa and DPI. ABA and GA_3_ contents were measured on the third day after seed incubation. Data are means ± SD (3 replicates × 30 seeds). The different letters denote a significant difference (*p* < 0.05) according to Tukey’s test.

As well as H_2_O_2_, CaCl_2_ induced seed germination, ABA decrease, GA increase, and decrease of ABA/GA ratio under ABA stress (Figure 5). To further investigate the role of H_2_O_2_ in Ca^2+^-induced seed germination and ABA/GA_3_ balance under ABA stress, DPI, an inhibitor of NADPH oxidases, was used to inhibit H_2_O_2_ production. DPI attenuated or completely abolished Ca^2+^-induced seed germination, ABA decrease, GA increase, and decrease of ABA/GA ratio under ABA stress.

**Figure 6:**
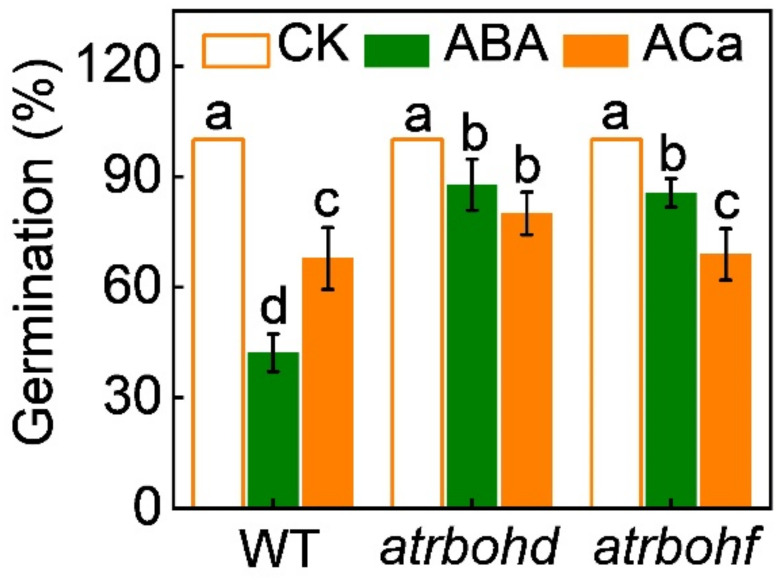
The effects of ABA and combination of ABA and CaCl_2_ (ACa) on the germination rates of seeds in wild-type (WT) *Arabidopsis* and *atrbohd* and *atrbohf* mutants. Germination rates were recorded on the seventh day after seed incubation. Data are means ± SD (3 replicates × 30 seeds). The different letters denote a significant difference (*p* < 0.05) according to Tukey’s test.

When compared to wild-type *Arabidopsis* seeds, seeds of the *atrbohd* and *atrbohf* mutants showed higher germination rates under ABA stress (Figure 6), suggesting that H_2_O_2_ is involved in ABA-induced inhibition of seed germination. However, the deletion of *AtRBOHD* and *AtRBOHF* completely abolished CaCl_2_-induced seed germination under ABA stress. Taken together, these results indicated that the Ca^2+^ and H_2_O_2_ signals interacted with each other, forming a reciprocal positive-regulatory loop, which antagonize ABA to promote seed germination.

## 4. Discussion

ABA and GA are two critical plant hormones and function antagonistically in regulating seed germination [7]. In the seeds exposed to salt and cold temperature, ABA accumulation was increased but GA_3_ accumulation was not decreased, indicating that ABA increase is the principal factor that disturbs the balance between ABA and GA_3_ and consequently inhibits seed germination unfavorable conditions [30].

As an important signal molecule, ROS generated by *RBOHs*, which encode NADPH oxidase, plays a dual role in regulating seed germination [30,34]. The H_2_O_2_ levels transiently increase during seed germination and inhibition of H_2_O_2_ production by NADPH inhibitor DPI or *AtRBOHD* deletion suppresses seed germination [35], suggesting that transient H_2_O_2_ generation is required for seed germination [14]. However, *atrbohd* and *atrbohf* double mutations impair ABA-induced promotion of ROS production and inhibition of seed germination, indicating that H_2_O_2_ was involved in ABA-induced seed dormancy [36].

In the present study, we found that exogenous H_2_O_2_ alleviated ABA-induced inhibition of seed germination, providing evidence on H_2_O_2_-ABA antagonism in regulating seed germination (Figure 1 and Figure 3). However, ABA pretreatment increased H_2_O_2_ accumulation and the transcript levels of *RBOHD* and *RBOHF* in melon seeds and *Arabidopsis* seeds with *AtRBOHD* or *AtRBOHF* deletion showed less sensitivity to ABA, indicating that H_2_O_2_ is required for ABA-induced seed dormancy (Figure 4 and Figure 6) [36]. In addition, the uncontrolled accumulation of H_2_O_2_ under stress conditions or during seed aging also inhibits seed germination [35]. Therefore, the occurrence of seed germination progresses is restricted to a critical range of H_2_O_2_ levels [34]. H_2_O_2_ at low or high levels would not permit progress toward germination. The previous study by Liu et al. showed that exogenous H_2_O_2_ promote ABA catabolism and GA biosynthesis during *Arabidopsis* seed imbibition [37]. Consistently, we found that exogenous H_2_O_2_ decreased the ABA level and increased the GA_3_ level during seed germination under ABA stress, suggesting that H_2_O_2_-induced seed germination was closely associated with its regulatory role in balancing ABA/GA_3_ (Figure 3).

Similar to H_2_O_2_, Ca^2+^ has a critical secondary signaling molecule and functions synergistically or antagonistically with ABA signaling during seed germination. For example, the repression of *Arabidopsis GLR3.5* (*AtGLR3.5*)*,* which encodes a plasma membrane Ca^2+^-permeable channel, impairs [Ca^2+^]_cyt_ elevation and enhances seed sensitivity to ABA, whereas *AtGLR3.5* overexpression promotes seed germination but reduce seed sensitivity to ABA by suppressing the expression of *ABSCISIC ACID INSENSITIVE4*, suggestive of a negative role of Ca^2+^ in ABA signaling [38]. However, the deletion of *TPC1* (a vacuolar Ca^2+^ channel gene) and Ca^2+^ signaling-related genes (e.g., *calcium-dependent protein kinase* *(CPK) 4*, *CPK11*, and the *calmodulin-like protein 39*) results in insensitivity to ABA during seed germination, suggestive of a positive role of the Ca^2+^ signal in ABA signaling [39,40,41].

In the current study, we found that CaCl_2_ pretreatment decreased the ABA/GA_3_ ratio likely by promoting ABA catabolism and GA_3_ biosynthesis and thus promoted seed germination under ABA stress, suggesting that cytoplasmic Ca^2+^ signaling acts as a positive regulator in ABA-regulated seed germination. Therefore, the Ca^2+^ signal plays a dual role in seed germination response to ABA as well as H_2_O_2_. Extracellular Ca^2+^ enters the cytosol through plasma membrane Ca^2+^-permeable channels to positively regulate seed germination, whereas the Ca^2+^ release from the vacuole through vacuolar channels is required for ABA signaling that inhibits seed germination.

The crosstalk between the H_2_O_2_ derived from RBOHs and the Ca^2+^ signal is well documented in various physiological actions [22]. Evidently, the H_2_O_2_-triggered influx of Ca^2+^ has long been thought to be involved in H_2_O_2_ sensing and signalling [42]. Ca^2+^ acts as a key downstream component of AtRBOHD and AtRBOHF, transducing ROS signals in plant growth and in responses to various stresses [36,43,44,45]. For example, in ABA signalling in guard cells, H_2_O_2_ activates Ca^2+^-permeable channels and induces influx of Ca^2+^ and increases in [Ca^2+^]_cyt_ in guard cells, which mediate stomatal closure induced by ABA [46,47]. However, the crosstalk between H_2_O_2_ and Ca^2+^ in regulating seed germination remains largely unknown. In the current study, H_2_O_2_ promoted Ca^2+^ influx in melon seeds under ABA stress, while the H_2_O_2_ deficiency in both melon and *Arabidopsis* seeds prevented Ca^2+^ influx under ABA stress (Figure 2). Similarly, the *atrbohd* and *atrbohf* mutations impair the hypoxia-elicited Ca^2+^ enhancement *in Arabidopsis* root cells [48]. CNGC20 is an important Ca^2+^ transport system that conducts external Ca^2+^ in the cytoplasm [49]. H_2_O_2_ induced the upregulation of *CmCNGC20,* suggesting that *CmCNGC20* may be involved in H_2_O_2_-induced Ca^2+^ influx (Figure 2B). Moreover, blocking of Ca^2+^ influx by LaCl_3_ or chelation of Ca^2+^ by EGTA inhibited H_2_O_2_-induced ABA/GA_3_ balance and germination in melon seeds pretreated with ABA (Figure 3A–C). *AtCNGC20* deletion in *Arabidopsis* seeds completely abolished H_2_O_2_-induced seed germination under ABA stress (Figure 3D). These results indicate that a Ca^2+^ signal is involved in H_2_O_2_-induced ABA/GA_3_ balance and subsequent seed germination under ABA stress.

Calcineurin-B-like (CBL) proteins and their interacting protein kinases (CIPKs) have been shown to function in many Ca^2+^-signaling processes, including seed germination. The mutations of *CBL9* and *CIPK3* exhibited hypersensitivity to ABA during seed germination, suggesting a negative role of Ca^2+^ signals in ABA-inhibited seed germination [50,51]. Interestingly, a calcium signal-activated CBL1/9-CIPK26 module can enhance ROS production via phosphorylation of RBOHF [52]. CPK5 phosphorylates RBOHD and produces ROS to induce systemic defense responses [53]. Here, CaCl_2_ increased H_2_O_2_ accumulation under ABA stress, accompanied by the upregulation of *CmRBOHD* and *CmRBOHF* (Figure 4).

Additional experiments showed that DPI prevented the CaCl_2_-induced ABA/GA_3_ balance and subsequent germination of melon seeds under ABA stress (Figure 5). Moreover, *AtRBOHD* and *AtRBOHF* deletion in *Arabidopsis* seeds completely abolished CaCl_2_-induced seed germination under ABA stress (Figure 6). Therefore, it is apparent that H_2_O_2_ participates in Ca^2+^-induced ABA/GA_3_ balance and subsequent seed germination under ABA stress. Taken together, the H_2_O_2_ and Ca^2+^ signals function together in a self-amplifying feedback loop, in which H_2_O_2_ induces Ca^2+^ influx, and Ca^2+^ subsequently increases H_2_O_2_ accumulation during seed response to ABA. Such an H_2_O_2_/Ca^2+^ activation circuit has also been reported to be required for rapid defense signal propagation in plants [53,54].

## 5. Conclusions

To date, the crosstalk underlying H_2_O_2_ and Ca^2+^ signals antagonize ABA to promote seed germination are unclear. In this study, we show that H_2_O_2_ and Ca^2+^ signals interact with each other to regulate ABA/GA_3_ balance and seed germination during ABA response. H_2_O_2_ promotes Ca^2+^ influx, which in turn increases H_2_O_2_ accumulation, forming a reciprocal positive-regulatory loop, to sustain Ca^2+^ influx-elicited signature and regulate ABA and GA_3_ balance. To our knowledge, this is the first study of its kind to provide evidence for the role of ROS and Ca^2+^ signaling in antagonizing ABA to regulate seed germination.

## Data Availability

All datasets generated for this study are included in the article.
